# The use of formal care for dementia from a professional perspective: a scoping review

**DOI:** 10.1186/s12913-022-08229-2

**Published:** 2022-06-25

**Authors:** Stefanie Bergmann, Julia Peper, Anja Bieber

**Affiliations:** Institute of Health and Nursing Sciences, Martin Luther University Halle-Wittenberg, Halle (Saale), Germany

**Keywords:** Access, Health and social care professionals, Dementia, Formal care, Influencing aspects

## Abstract

**Background and objectives:**

The progressive character of dementia usually leads to a continuously increasing need for support. There is some evidence of late use of professional support during the disease course. We aim to provide an overview of aspects influencing access and use of formal care in dementia from the perspective of health and social care professionals. Additionally, the perspectives of professionals and people with dementia/informal carers will be compared.

**Methods:**

We conducted a scoping review with a systematic literature search in Medline via Ovid in January 2019 and updated this in April 2020 and in May 2021. Publications were considered eligible when focusing on influencing aspects of the use of formal care or support for people with dementia in an outpatient setting from the perspective of health professionals. Included publications were critically appraised using the Mixed Method Appraisal Tool. We identified aspects of access to and use of formal care and support services. A consultation exercise with three specialised trained dementia care nurses was conducted to validate our results.

**Results:**

We included 29 studies: *n* = 20 qualitative, *n* = 6 quantitative-descriptive, *n* = 3 mixed-methods. Various support services were identified, but a focus was on services for diagnostic and treatment of dementia. A wide range of influencing aspects (*n* = 15) describe the access to and use of formal care services. Aspects related to the complexity and structure of the healthcare system and the competence of professionals were frequently addressed. Second, attitudes and expectations of professionals, and experiences with people with dementia and their informal carers were identified. The dementia care nurses highlighted the importance of coordinated care to enhance dementia-specific competencies.

**Conclusions:**

Health and social care professionals still describe barriers in accessing and using formal care due to various influences. Ways to improve access to and use of professional support in dementia should consider individual and system-level activities, as well as overarching aspects. Important topics are therefore education and training of professionals and coordinated dementia-specific care to provide adequate support for people with dementia and their relatives. Several professions may be involved in this increasingly important field, e.g., nurses with a dementia-specific training like dementia care nurses.

## Background

Dementia is a syndrome resulting from a progressive or chronic disease of the brain that severely impairs its cortical functions [1]. The symptoms that progress over time, such as memory loss, loss of orientation and communication, impaired decision-making ability, and the gradual decline in activities of daily life result in a significantly increased need for care and support [2, 3]. Nevertheless, professional care or support services are used rather late in the course of the disease [4]. Dementia care is mainly provided by family members, e. g. spouses or children [4–6]. This might be due to the fact that people with dementia often refuse formal support, or informal carers may wish to provide care themselves because of inner motivations (e. g. beliefs, values, characteristics). Another common reason is the lack of information provided by professionals regarding the access to available services. In addition, there are also many systemic barriers or sociodemographic hindrances, such as living in a rural area, which result in care services not being used or often being used late in the course of the dementia [7].

These problems have been addressed by the Actifcare project (Actifcare = Access to timely formal dementia care in Europe), where the access to and use of formal care by people with dementia and their informal carers was examined [8]. A recent review [7] investigated influencing aspects on the access to and utilization of formal care from the perspectives of people with dementia and their informal carers. This review indicated a closer examination of professionals in the healthcare system [7]. Therefore, we conducted a scoping review to examine the perspectives of healthcare professionals.

### Objectives

The aim of this scoping review is to provide an overview of the investigated aspects influencing access to and use of formal care and support for dementia from the perspective of health and social care professionals. The second aim is to compare the perspectives of the professionals with the perspective of people with dementia and informal carers.

## Methods

### Design

The scoping review design was chosen because it is suitable for providing an overview of related investigations and for informing decision makers as well as researchers about gaps in the topic.

We used the methodology used by Khalil et al. [9]. This methodology is based on the framework by Arksey and O'Malley [10], the basic methodological approach for scoping reviews. Studies with different designs can be included in a scoping review.

In contrast to systematic reviews, a quality appraisal of the included studies is not foreseen in a scoping review [10, 11] and most often not carried out [12]. Since some methodological literature suggest critical appraisal in order to enrich the meaningfulness of the analysis [13], we decided to include a critical appraisal using the validated Mixed Method Appraisal Tool (MMAT) [14].

### Search methods

A systematic literature search in the database Medline via Ovid was initially conducted in January 2019 and updated in April 2020 and in May 2021. No time limitations were set. We used the following search terms: dement*, Alzheimer*, care giving, professional care, formal care, care, health services, health care, social care, home care, community care, long-term care, formal support, Delivery of Health Care, Healthcare Disparities, Health Services Accessibility, service use*, utilisation, utilization, access*, service use, service non-use*, help-seeking, help seeking, health services misuse and health services needs and demand.

### Inclusion criteria

Publications were included if they dealt with influencing aspects in the use of formal support for adults with dementia in an outpatient setting. In addition, the issue had to be examined from the perspective of professionals. Formal care/help is defined as outpatient care, which includes health and social services such as home care services, counselling, long-term and day care. Care services must also be provided by a professional, paid specialist. All study designs and English or German language publications were considered as eligible.

Publications were excluded that referred to individuals with suspected dementia or without a dementia diagnosis. This also applied to diagnoses of mild cognitive impairment, postoperative delirium, any form of amnesia or Korsakov syndrome. Other exclusion criteria were studies addressing palliative support services and focussing exclusively on the perspective of informal carers as well as on the people with dementia.

### Study selection

Titles and abstracts were screened independently by two reviewers (SB & JP). Conflicts were resolved by discussion. If no consensus was reached, the third reviewer (AB) was consulted to achieve agreement.

The full-text screening was carried out by two reviewers (SB & JP) using a screening checklist. The third reviewer (AB) supported in case of disagreement. After the screening, the references of the included articles were additionally checked for further suitable publications to ensure that no relevant articles were overlooked. 

### Analysis

An appropriate data extraction strategy was developed in advance. Based on Bieber et al. [7], the following points were recorded: study design, study location, study population, severity of dementia, theoretical framework used and formal care services examined. In addition, the methodological approach, number of participants, target dementia population and information on the methodological approach were extracted.

The results were examined regarding influencing aspects. Firstly, two reviewers (SB & JP) separately reduced the material of the study findings to information regarding influencing aspects. Afterwards, they compared and discussed their results. All the influencing aspects were narratively described. To determine similarities and differences between the professional perspective and the perspective of people with dementia and their informal carers, the results of the scoping review by Bieber et al. [7] were used.

### Consultation exercise

In order to achieve further valuable insights, we conducted a consultation exercise. This methodological step is described by Arksey and O’Malley [10] as a way to validate the findings of a scoping review.

We conducted an online meeting with three trained dementia care nurses to discuss the results of our scoping review. The aim was to debate the results with experts in the field of dementia care consultation. The dementia care nurses were members of a project team at the Martin Luther University Halle-Wittenberg in Germany. In the project, people with dementia and their relatives received information, advice and support to enable them to lead a self-determined life at home [15]. The dementia care nurses were trained in dementia care and case management. The participating nurses received the results of the scoping review beforehand the meeting. The meeting lasted 90 min.

## Results

**Fig. 1:**
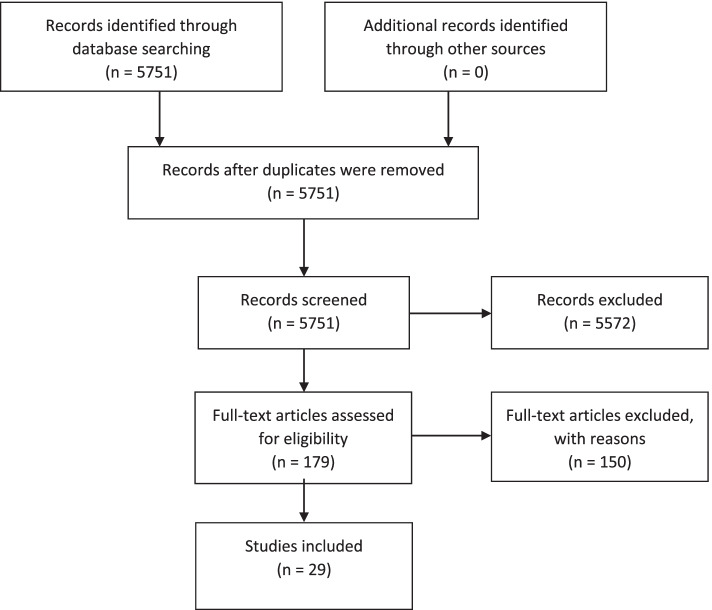
Flowchart of the selection process

A total of 29 studies were included. Figure 1 shows the full selection process.

### Overview of the characteristics of the included studies

Table 1 shows the characteristics of the included publications.

**Table 1:** Overview of the characteristics of the included studies

Author	Year	Country	Study type	Number of participants	Professions	Target dementia population	Services
Berdai Chaouni et al. [37]	2019	Belgium	Qualitative	*n* = 13	Psychologists, neurologists, general practitioners, intercultural mediators, head nurses of geriatric/dementia department, nurse s& social nurses, rheumatologists/revalidation doctors	People with dementia with a Moroccan migrant background	3
Bisset et al. [25]	1996	UK/Scotland	Quantitative descriptive study	*n* = 241	General practitioners	People with dementia living in the community	1, 3
Blix et al. [28]	2017	Norway	Qualitative	*n *= 18	Registered nurses, licensed practical nurses	Indigenous people of Scandinavia with dementia	Various
Bourqe & Foley [26]	2020	Ireland	Qualitative	*n* = 12	General practitioners	People with dementia, not specified	1
Bowes et al. [34]	2003	United Kingdom	Qualitative	*n* = 11	General practitioners, community psychiatric nurses, consultants, National Health Service (NHS) ethnic minority health projects, voluntary sector providers of services for older people and people with mental health problems from minority ethnic groups	Asian people with dementia living in Scotland	1, 3, 8
Brijnath et al. [41]	2021	Australia	Qualitative	*n *= 27	Service providers in social and clinical care	People with dementia of minority ethnic groups in urban areas in Australia	Various
Broda et al. [31]	2017	Germany, Ireland, Italy, Netherlands, Norway, Portugal, Sweden, United Kingdom	Qualitative	*n *= 38	Policy makers (elected), representatives of ministries or governmental departments in permanent positions, representatives of relevant non-governmental organisations, Alzheimer societies or umbrella organizations providing formal dementia care	People with dementia, not specified	1–6, 10
Cheung et al. [38]	2019	New Zealand	Qualitative	*n* = 11	Nurses, social workers, occupational therapists, community support workers, community advisors	Asian people with dementia living in New Zealand	Various
Constantinescu et al. [21]	2018	Canada	Qualitative	*n* = 16	Family physicians	People with dementia living in rural communities	1
Cording et al. [22]	2017	Germany	Quantitative	*n* = 177	General practitioners and different specialists	People with dementia living in the community	1
Dal Bello-Haasm et al. [33]	2014	Canada	Mixed methods	*n* = 16	Healthcare providers, physicians, non-physicians, family physicians	People with dementia living in rural areas	1–4, 8, 10
Franz et al. [18]	2010	USA	Qualitative	*n* = 40	Primary care physicians	Primarily managed care around a large urban city	1, 2
Giezendanner et al. [23]	2018	Switzerland	Quantitative	*n* = 882	General practitioners	People with dementia, not specified	1
Gulestø et al. [42]	2020	Norway	Qualitative	*n* = 13	Registered nurses, qualified nursing assistants, occupational therapists	Family carers from minority ethnic groups caring for people with dementia in Norwegian communities	3
Hansen et al. [27]	2017	Norway	Qualitative	*n* = 24	Registered nurses, assistant nurses	People with dementia, not specified	1
Haralambous et al. [36]	2014	Australia	Qualitative	*n* = 55	General practitioners, geriatricians, psychiatrists, practice nurses, herbalists, residential aged care staff (physiotherapist, nurses and personal care attendants), pharmacists, community workers	Asian people with dementia living in Australia	1–4, 6, 8, 10
Hinton et al. [17]	2007	USA	Qualitative	*n* = 40	Primary care physicians	People with dementia, not specified	1–5, 7, 9
Hum et al. [24]	2014	Canada	Qualitative	*n* = 12	Family physicians, a neurologist, geriatricians, and geriatric psychiatrists	People with dementia living in the community	1
Jansen et al. [32]	2009	Canada	Qualitative	*n* = 44	Home care nurses, home care aides, therapists, administrators, care coordinators	People with dementia living in rural and urban areas	1–8
Kosteniuk et al. [16]	2014	Canada	Qualitative	*n* = 15	Family physicians	People with dementia living in the rural areas	1, 3, 5
Lange et al [43]	2018	Netherlands	Qualitative	*n* = 146	Case managers, general practitioners, general practice nurses, neurologists, geriatricians, nurses, nursing assistants, project-leaders, psychologists, managers, lobbyists, welfare policymakers	People with dementia, not specified	3
Nielsen et al. [30]	2019	Denmark	Quantitative descriptive study	*n* = 47	Primary care dementia coordinators	People with dementia of minority ethnic groups in Denmark	5
O’Connor et al. [29]	2020	Australia	Qualitative	*n* = 2	Aged and community-care providers	People with dementia, not specified	1, 3, 5
Stephan et al. [35]	2015	Germany	Qualitative	*n* = 6	Nurses, social workers, geriatricians, psychologists, members of a municipality	People with dementia, not specified	3–7
Stephan et al. [6]c	2018	Germany, Ireland, Italy, Netherlands, Norway, Portugal, Sweden, the United Kingdom	Qualitative	*n* = 144	Registered, assistant and community mental health nurses, social workers, general practitioners, other specialist physicians, psychologists, counsellors’ educators, case managers	People with dementia, not specified	1, 3–7
Stolee et al. [40]	2021	Canada	Mixed methods	*n* = 33	Health care providers, health care administrators, Represent several sectors (family health teams, behavioural health services, long-term care, memory clinics, the SW LHIN, and several community-based organizations) and policy makers	People with dementia and their care partners in rural communities	Various
Werner [19]	2007	Israel	Quantitative descriptive study	*n* = 395	Family physicians	People with dementia, not specified	Various
Williams [20]	2000	United Kingdom	Mixed methods	*n* = 118	General practitioners	People with dementia, not specified	1–5
Wyman [39]	2021	USA	Quantitative descriptive study	*n* = 65	Social workers, nurses, psychologists, clinical pharmacists, peer supporters, psychiatrists, physicians and other professionals (not specified)	People with dementia, not specified	1

Formal care and support services: 1 Diagnostic and treatment, 2 Counselling, education, information, 3 Specialized dementia care, 4 Domestic support, 5 Coordinated care, 6 Inpatient care, 7 Semi-inpatient care, 8 Culturally sensitive services, 9 Self-help offerings, 10 Public education

The studies involved professionals from various professional backgrounds. Eleven of these studies focused [16–26] on the perspective of physicians (predominantly primary care physicians) and two studies [27, 28] on the perspective of nurses (registered nurses, licensed practical nurses and assistant nurses). One study interviewed providers of care for the elderly [29] and another one examined the perspective of primary care dementia coordinators [30]. The remaining fourteen studies [6, 31–43] included various professions without differentiating between them. Most of these references also included physicians [6, 33–35, 37, 39, 40, 43] and various nurses [6, 31, 32, 34, 35, 37–39, 42, 43].

Most of the studies did not refer to a theoretical framework [6, 16–20, 22–35, 37–39, 41, 43]. Only four studies [21, 36, 40, 42] reported to have used a theoretical framework. Haralambous et al. [36] used the *Cultural Exchange Model.* This model is a framework that focuses on understanding knowledge development as an iterative process of exchange between researchers and stakeholders [36]. A Canadian study by Constantinescu et al. [21] is based on the *Theoretical Domains Framework*, which is a validated framework with a consensus approach. It includes 14 theoretical domains and is intended to categorize applicable psychological theories [21]. In the qualitative study by Stolee et al. [40], researchers developed a framework they called ‘*Whole Person, Whole Journey*’ to develop a national dementia strategy for the rural area of southwestern Ontario, Canada. This framework is based on the experiences and perspectives of care providers and administrators as well as people with dementia and their family carers with regard to the strengths and gaps in dementia care within the local health care system. The Norwegian study by Gulestø [42] et al. uses a structural approach based on the theory of sociologist Pierre Bourdieu. The theory intended to serve as a ‘critical and reflexive lens’ through which perceptions and understandings of healthcare professionals may be better grasped in relation to dementia care.

We defined ten categories of different services (Table 1) of formal care and support services: the category of *diagnostics and treatment* (1) includes the consultation with specialists as well as specific diagnostic procedures and a variety of specific therapeutic approaches. *Counselling, training and information* (2) highlights services aiming at counselling or training people with dementia or their informal carers, along with offers of information. The category *specialising of care and social services in dementia* (3) includes the need for specifically trained staff from care or social services, for example dementia care nurses or community workers. *Domestic support* (4) includes all services that provide support for people with dementia in their households and relieve the burden on their family members. *Coordination of care* (5) is defined by collaborative approaches between different service providers or by persons or institutions networking and coordinating care. *Inpatient care* (6) includes, for instance, offers such as short-term care, hospital stays as well as long-term care in nursing homes. *Semi-inpatient care* (7), on the other hand, involves night care and day care services, among others. *Culturally sensitive services* (8) aim at addressing the specific needs of minorities both in culture and in language. In addition to *self-help offers* (9) the included publications also called for enhancing *public education* (10), for example with public campaigns.

### Critical appraisal

The results of the critical appraisal of the included studies with the MMAT measurement are presented in Table 2**.** More than 80% of the qualitative studies fulfilled all the criteria. The reporting quality of the six quantitative studies was moderate. The three mixed-methods studies achieved between three to six of the seven criteria.

**Table 2:** Internal validity according to MMAT

Study Type	MMAT item	3	Can’t tell	No
Qualitative (*n* = 20)	S1. Are there clear research questions?	20		
	S2. Do the collected data allow to address the research questions?	20		
	1.1. Is the qualitative approach appropriate to answer the research question?	20		
	1.2. Are the qualitative data collection methods adequate to address the research question?	18	2	
	1.3. Are the findings adequately derived from the data?	17	3	
	1.4. Is the interpretation of results sufficiently substantiated by data?	18	2	
	1.5. Is there coherence between qualitative data sources, collection, analysis and interpretation?	20		
Quantitative (*n *= 6)	S1. Are there clear research questions?	5	1	
	S2. Do the collected data allow to address the research questions?	5	1	
	4.1. Is the sampling strategy relevant to address the research question?	4	2	
	4.2. Is the sample representative of the target population?	5	1	
	4.3. Are the measurements appropriate?	6		
	4.4. Is the risk of nonresponse bias low?	2	1	3
	4.5. Is the statistical analysis appropriate to answer the research question?	5	1	
Mixed methods (*n* = 3)	S1. Are there clear research questions?	2		
	S2. Do the collected data allow to address the research questions?	2		
	5.1. Is there an adequate rationale for using a mixed methods design to address the research question?	2		1
	5.2. Are the different components of the study effectively integrated to answer the research question?	2	1	
	5.3. Are the outputs of the integration of qualitative and quantitative components adequately interpreted?	2	1	
	5.4. Are divergences and inconsistencies between quantitative and qualitative results adequately addressed?	1	2	
	5.5. Do the different components of the study adhere to the quality criteria of each tradition of the methods involved?	1	1	1

### Aspects of access to and use of formal care services

We identified 15 aspects that could influence access to and use of formal care services. We mapped the identified aspects into three global themes: 1) *Aspects relating to the individuals involved*, 2) *Aspects relating to the health and social care systems*, and 3) *Overarching aspects*. The topic *Aspects relating to the individuals involved* was divided into three sub-themes. Table 3 displays an overview of all these aspects. Each description of the identified aspects ends with selected results from the consultation step with the dementia care nurses.

**Table 3:** Overview of the access to and use of formal care services

Global themes and subthemes	References
**Aspects related to the individuals involved**	
**People with dementia and their informal carers**	
Ethnicity	[28, 30, 31, 33, 34, 36, 37, 41, 42]
Region of residence	[16, 21, 23, 26, 29, 32, 33]
Attitudes, expectations and experiences towards formal care and dementia	[6, 20, 21, 30, 34, 35, 37, 38, 42]
Family situation and social background	[6, 18, 21, 30, 32, 35, 36]
**Professionals**	
Competence of the professionals	[6, 17–20, 24, 29, 30, 32, 34–37, 39, 42, 43]
Time resources of physicians	[17, 18, 24, 32]
Perceptions and attitudes of the healthcare professionals	[6, 21, 27, 30, 35]
**Professionals, people with dementia and their informal carers**	
Relationship between professionals and people with dementia and their family	[6, 24, 27, 29, 32, 35]
**Aspects related to the health- and social care systems**	
Structures and complexity of the healthcare system	[6, 16–18, 20, 23, 25, 26, 29, 31, 32, 34–36]
Financial aspects of the healthcare system	[17, 21, 29, 31, 32, 35]
Multi-professional and interdisciplinary cooperation between institutions, service providers and professionals	[18, 21, 23–25, 29, 30, 35, 43]
Coordinating care by persons or institutions	[6, 16, 22, 24, 28, 29, 31, 32, 35, 40]
**Overarching aspects**	
Information about dementia and support services	[6, 29–33, 36, 42]
Stigmatization and public awareness	[6, 20, 26, 29, 31, 33, 34, 36, 40, 41]
Early planning of formal care	[6, 20, 26, 35]

### Aspects relating to the individuals involved

#### People with dementia and their informal carers

##### Ethnicity

Ethnic aspects were addressed in eight studies as possible barriers to using care services [28, 30, 31, 33, 34, 36, 37, 41, 42]. Those barriers included family obligations, language barriers, and the lack of culture- and religion-sensitive support services. Informal carers from ethnic minorities in Australia, Denmark and Norway undertake more family obligations to care for a relative with dementia without professional support than other informal carers [28, 30, 41]. Language barriers were described for informal carers from ethnic minorities in Australia, Norway, and the United Kingdom [28, 34, 36, 41, 42]. Better translation services and culturally more appropriate assessment tools for non-English speakers (or non-native speakers) and their families were demanded in a Canadian study [33]. People with dementia in Belgium, who had a Moroccan migrant background, rejected support services, because of a lack of culture- and religion-sensitive service offers [37]. Intercultural mediators might help to overcome such barriers [37].

The dementia care nurses supported only a few people with dementia with a migration background. They assume that access to formal care for people from ethnic minorities was more difficult in the rural region of Saxony-Anhalt in Germany than elsewhere.

### Region of residence

Seven studies [16, 21, 23, 26, 29, 32, 33] described regional influences on the use of care services, depending on where the people with dementia lived. The availability of services is mentioned as an important difference between rural and urban areas. GPs from Germany and Switzerland reported a lack of services in rural regions [16, 23]. Canadian people with dementia living in rural areas were often faced with the lack of governmental and/or personal resources for traveling to healthcare facilities [33]. GPs in Ireland highlighted the need for uniform access to care regardless of region [26]. Appropriate support services were reported in urban areas of Canada [21, 32], Switzerland [23], and Australia [29]. Canadian rural family physicians had varying opinions, i.e. some felt that people with dementia had good access to services, while others disagreed [21]. Living in a rural region could also have supporting aspects such as better social relationships between healthcare providers and the families as well as closer social integration in the community [16].

The dementia care nurses confirmed this finding from their own experience and mentioned the varying number of care providers in urban and rural areas in Saxony-Anhalt, with the consequence that some offers in rural areas were too far away for the people with dementia.

### Attitudes, expectations and experiences of informal carers and people with dementia towards dementia and formal care

*Attitudes and beliefs of informal carers and people with dementia towards the diagnosis of dementia and towards the disease itself* might be a significant aspect influencing the use of formal care [6, 20, 34, 37, 38, 42]. People with dementia or informal carers could have problems accepting the diagnosis and thus they do not accept or seek help [6, 20, 37, 42]. Several professionals stated that, in the case of some informal carers with a migrant background, informal carers were unable to recognise or accept the diagnosis of dementia or they even felt ashamed of their relative with dementia and hid the diagnosis or even isolated the family member with dementia from social interacting [34, 38].

In the experience of dementia care nurses, people with cognitive impairment or their relatives or both needed time to accept the diagnosis of dementia.

Moreover, *expectations, attitudes and beliefs of informal carers and people with dementia regarding formal services or/and professionals* might also influence service use [6, 30, 34, 35, 37]. For example, a strong sense of duty to take care of the relative with dementia without professional help might be a barrier to using care services [6, 35, 37]; the same applies to unrealistic expectations of formal services [21]. The expectations might be high during a crisis, but the scope for action by healthcare professionals is limited [35]. Strong emotions of informal carers of people with dementia, such as fear or anxiety (e.g. the fear of being separated from their relative with dementia), were also a contributing aspect to not seeking or even rejecting formal care [6]. Only a few South Asian people in the UK used care services, because they believed dementia is not a specific disease, but simply a problem of old age [34]. The use of formal care might influence the further use of other services. Experiences of informal carers and people with dementia towards dementia and formal care could also influence service use [6, 34]. Positive experiences might encourage people with dementia and/or their informal carers to continue using services [6] while negative experiences with formal care services might inhibit further use [34].

Dementia care nurses experience suggested that professionals should explain the options available from formal care services and provide information about the consequences of the various options.

### Family situation and social background

The *family situation and social background* was mentioned in seven studies [6, 18, 21, 30, 32, 35, 36]. Primary care coordinators from Denmark found it generally difficult to make contact with the families, who often preferred to take care of their elderly family members themselves [30]. The existence of a social network, enabling the distribution of care responsibilities, could stabilise the care situation at home and delay the use of services [6]. Family physicians from Canada described that caring for a relative with dementia might produce negative emotions, such as distress and frustration. Such emotions might also be a barrier to dementia care [21]. Family conflicts might be a barrier to searching for help but on the other hand they might trigger the use of formal care services [6, 36]. Disagreements in the family about treatment options might also influence the utilization of referrals to mental health specialists [18].

Financial circumstances of the families might influence service use [6, 32]. For example, due to financial reasons, people with dementia and informal carers might be reluctant to accept care assistance for incontinence. Ultimately, this could lead to the need for more expensive services [32]. Furthermore, required private payments might also play a significant role, meaning that services had to be affordable [6].

The dementia care nurses mentioned a lack of social networks as a barrier to accessing support services. The influence of financial circumstances was not discussed by the dementia care nurses.

### Professionals

#### Competencies of the professionals

The competence of health-care professionals was mentioned in most of the included studies [6, 17–20, 24, 29, 30, 32, 34–37, 39, 42, 43]. Competencies were described as dementia expertise, social competencies, cultural care skills, and coordination skills.

A lack of dementia expertise was reported by family physicians [18, 21], professionals involved in dementia case management [43] and different healthcare professionals [34]. Professionals mentioned the need for education and training in dementia care and treatment [20, 29, 32, 34, 36]. In an Israeli study [19] the knowledge of Alzheimer symptoms was an advantage and helped to realize that assistance was needed.

Social competencies of the professionals were mentioned as important facilitators in cooperating with informal carers and in supporting the use of formal care, i.e. being proactive in seeking and maintaining contact [35]. Seeing people with dementia as challenging care recipients or the patronising behaviour of the professionals were seen as barriers to accessing appropriate help [6]. Professionals involved in Dutch dementia case management mentioned the importance of social competencies, which include the ability to reflect on their own behaviour and own limits [43]. Providers of geriatric mental health care for veterans highlighted the importance of effective communication with informal carers as well as the need for training in this issue [39].

In four studies it was found that it might be important for professionals to have some knowledge about the culture of people with dementia [30, 36, 37, 42]. One of these studies related to people with dementia from minority ethnic communities in Demark, i.e. first-degree descendants from the Middle East, immigrants from eastern Europe, Pakistan and others [30]. The other studies investigated dementia care aspects of families with Chinese and Vietnamese backgrounds in Australia [36], and of Moroccan migrants in Belgium [37]. A Norwegian study investigated perceptions of healthcare professionals of community-based dementia teams towards their roles in reaching and supporting informal carers from minority ethnic backgrounds [42]. Primary care dementia coordinators in Denmark mentioned, among other things, communication difficulties and inadequate sensitivity of care workers as barriers for the use of post-diagnostic services [30]. Australian health professionals believed that dementia specific education and information would help them to enhance access to services for people with an Asian background [36]. Professionals from different healthcare fields described the immense commitment of Moroccan informal carers to caring for their families and their own long-term health problems as a result of caregiving [37]. Some of the professionals were aware of these potential impacts and took action. However, they were concerned that not all colleagues would react to this problem [37]. The Norwegian dementia team members described that a number of distinct attributes are needed to navigate a complex dementia health-care system [42].

Coordinating skills were mentioned by several healthcare professionals [17, 19]. Primary care physicians considered that they might not have the time or knowledge to help families in accessing social services like meals on wheels, adult day care and in-home supportive services [17]. Family physicians were more likely to recommend seeking help from professionals than from non-professional sources. They were also more likely to recommend primary-care sources (especially general physicians and social workers) than other medical specialists, like psychiatrists or neurologists [19].

The dementia care nurses emphasized that every professional working in the care of people with dementia needed dementia-specific health and social competencies.

### Time resources of physicians

Some studies highlighted the influence of the *time resources of physicians* on the access and use of formal dementia care [17, 18, 24, 32], as these might be insufficient [17, 18, 24]. The health needs of people with dementia might be too complex to be comprehensively treated in the normal treatment time [17]. Due to their limited short-term memory, it is necessary to involve relatives. Overall, this results in an ethical dilemma, since involving relatives in the visit to the physician is important but very time consuming [17]. Jansen et al. [32] argued that long waiting lists and waiting times would complicate access to and utilisation of formal care. Additional time and workload through the difficulties in access to and use of referrals for patients with dementia and neuropsychiatric symptoms contribute to burn out, lack of control and powerlessness of primary care physicians [17, 18, 24]. Physicians who were unfamiliar with community services had neither the time nor the interest to engage with them [17, 18, 24].

The dementia care nurses pointed out that professionals other than physicians sometimes have more contact with people with dementia and informal carers and these could therefore help to improve access to formal care.

### Perceptions and attitudes of the healthcare professionals

P*erceptions and attitudes of healthcare professionals* towards people with dementia might have an influence on the use of care or support [6, 21, 27, 30, 35]. Primary care dementia coordinators in Denmark mentioned that the attitude of professionals toward minority ethnic service users might be obstructive [30]. The attitudes of healthcare professionals towards people with dementia can be a hindrance as well as a facilitator [6]. For example, patronising behaviour by healthcare professionals might be a barrier. Respect for people with dementia, paying attention to their capabilities and their rights and needs might be a facilitator for the use of formal care. Healthcare professionals regarded informal carers as responsible for finding formal care or support, but sometimes informal carers thought that they should wait to be addressed by professionals (e.g. physicians) [35]. Nurses in a US study mentioned psychosocial needs as important basic needs of people with dementia, but priority in professional dementia care was still given to physical needs [27]. Constantinescu et al. [21] reported that physicians in rural areas felt that it was often their responsibility to care for people with dementia in their community.

The dementia care nurses were unsure whether professionals were taking into account the whole situation of people with dementia and their informal carers, or whether they were only focussing on their professional tasks.

### Professionals, people with dementia and their informal carers

#### Relationship between professionals and people with dementia and their family

The relationship between healthcare professionals and both people with dementia and informal carers was highlighted in six publications [6, 24, 27, 29, 32, 35]. Nurses involved in a US study mentioned that it was helpful to know the people with dementia in order to identify their resources and opportunities for participation [27]. Important for a good cooperation between health professionals and the families were trustful relationships characterized by a proactive, early [35] and permanent contact [6]. Trusting relationships were regarded as facilitating the improved use of support services [6]. People with dementia and their relatives would need to be involved in all decision-making processes [6]. The findings of Jansen et al. [32] indicated that a lack of flexibility in care arrangements could preclude the development of a trusting relationship. In Denmark, physicians were responsible for reporting unsafe drivers to the Ministry of Transport. However, the physicians were not comfortable with this responsibility and pointed out that this could harm the relationship between people with dementia and physicians [24]. In an Australian study, care providers for older people and community care providers described partnerships with families as a strategy that support them in providing reablement interventions [29].

The dementia care nurses confirmed, that a relationship of trust between professionals and people with dementia and their informal carers is an essential prerequisite for accepting professional help. They highlighted that building a trusting relationship between people with dementia, informal carers and professionals is time-consuming.

### Aspects related to the health- and social care systems

#### Structures and complexity of the healthcare system

Structural aspects were mentioned in several studies, especially a lack of dementia specific support services, like memory clinics, nurses with dementia specific training, or dementia-specific community workers [16, 18, 20, 25, 26, 32, 36]. Primary physicians from Northern California mentioned few or unevenly distributed trained psychiatrists especially in rural areas [18]. General practitioners from Ireland demanded uniform access to care irrespective of geography [26]. Additional services were needed and requested, like memory training, vacation offers, day or night care or re-ablement interventions [23, 25, 29]. Moreover, the need for socio-emotional support services [6, 20, 31–35] and cultural-specific services [31, 34] was highlighted. Health-care experts from some European countries identified a lack of services for people with special needs, such as a low socio-economic status or people with early-onset dementia [31].

The complexity of the healthcare systems was described as a barrier for the use of formal care in dementia [6, 17, 18, 32]. An cross-European study noted the disjointed nature of the health care system(s), system inconsistencies, and service inequity across each country [6]. That causes a high degree of variability and unclear roles among health- and social care professionals. High bureaucratic hurdles [6, 17] as well as inappropriate time resources for the caregiving activities [32] were mentioned. Primary care physicians from the US reported complicated access to mental health care services [18].

The dementia care nurses explained that people with dementia, informal carers and even health-care professionals often have difficulties in understanding the complex structures of support systems and their financial aspects.

### Financial aspects of the healthcare system

*Financial aspects of the healthcare systems* were described several times as an important influence [17, 21, 29, 31, 32, 35]. Hinton et al. [17] reported that the anticipated remuneration would be a barrier, because it did not reflect the time-intensive nature of dementia care in the United States. A German study [35] showed that the competitiveness in the national health and long-term care systems could be a barrier. That would include the financial interests of care providers. Canadian family physicians described insufficient financing of home visits by rural practitioners [21].

Three studies addressed the *allocation of public funds and resources* [29, 31, 32]. A lack of funds and resources or limitations were found as important reasons for inadequate management of the complexity and continuity of dementia care in Australia [29], some European countries [31] and Canada [32]. Experts from various European countries suggested, the reallocation and reorganisation of funds and resources could increase efficiency and thus improve dementia care [31]. In the perspective of elderly and community care providers in Australia, the increased time spent caring for people with dementia would not be covered in the funding [29]. Fiscal resource allocation might be aligned with formal provider and informal carers needs [32].

The dementia care nurses described the difficulty of identifying financial resources and conditions for funding support services for people with dementia living at home. This sometimes led to an untimely transition to nursing homes.

### Multi-professional and interdisciplinary cooperation between institutions, service providers and professionals

*Multi-professional and interdisciplinary cooperation between health- and social care professionals* as well as different *institutions* facilitates access to formal services [18, 21, 23, 24, 29, 30, 35, 43]. Important moderators for good cooperation were defined aims, the personal relationship between health care professionals and the clear allocation of responsibilities [35]. Difficulties in communication between primary care physicians and specialists (e.g. neurologist) might be a hindrance in accessing and coordinating specialist care [18]. Swiss and Canadian physicians appreciated multidisciplinary teams [21, 23]. Good communication and collaboration with home care nurses, as well as between different physicians were seen as beneficial for dementia care [21]. Australian community care providers of re-ablement interventions described that limitations in referral pathways and increased competition between providers led to fewer collaborations between organisations [29]. For dementia care of people with migrant backgrounds it would be beneficial to consult colleagues from the same community [30]. Collaboration between physicians and members of the voluntary sector, such as the Alzheimer’s Societies, could enhance formal dementia care [24]. Dutch dementia case managers highlighted the required cooperation between health care professionals, especially between general practitioners and case managers, and between primary and secondary health-care professionals [43]. The dementia care nurses confirmed the need of cooperation between different health and social care organisations. However, they stated that health care professionals were often unable to think outside the box.

### Coordinating care by persons or institutions

Several studies described the benefit of a coordinating person (e.g. case manager) or institution that support people with dementia and their informal carers to find timely and appropriate health or social care [6, 16, 24, 28, 29, 31, 32, 35, 40]. Coordination could be performed by different professions such as case managers, geriatricians, general practitioners, dementia advisors, social workers, multidisciplinary teams or by a dementia link support worker [29, 31]. Canadian family physicians noted that they had a crucial role in caring for people with dementia and their informal carers. They had to provide counselling, education and links to community support services [24]. German physicians saw potential for providing support by establishing a professional contact point to which they could turn as healthcare providers in the case of specific issues [22]. Canadian health care administrators and policy makers recommended a system navigator or a care coordinator role in order to streamline processes [40].

The dementia care nurses emphasized the importance of clear agreements regarding responsibilities between health-care professionals or between several services.

### Overarching aspects

#### Information about dementia and support services

Seven publications addressed the need for *information,* either for professionals, people with dementia or for informal carers [6, 29–33, 42]. Access to information was seen as a first step in accessing support services [31]. Healthcare professionals across Europe called for sufficient, clear, understandable and precise information on dementia and emphasized the importance for information about available services and legal issues [6]. The European Actifcare study [31] stated that it would be beneficial for people with dementia and their relatives, if information on available service provision were available and accessible. Experts suggested an online platform or website that could inform people with a family member with dementia about available care services and support offers to enhance the access to information and available service providers [31]. An Australian publication stated that some health care organisations were taking advantage of education services and resources provided by branches of Dementia Australia [29]. The educational level of service users might have an influence on the use of formal services [30]. All of the professionals interviewed in a Norwegian study [42] reported positive experiences with educational courses for family members of people with dementia. It was found out that counselling services may be useful for people with memory issues [32, 33], as well as for informal carers [32].

There is a lot of information material available, but this often does not meet the needs of people with dementia or informal carers or take regional aspects into account was the opinion of dementia care nurses.

### Stigmatization and public awareness

*Stigmatisation or public awareness* were addressed in ten studies [6, 20, 26, 29, 31, 33, 36, 38, 40, 41]. Dementia is often still considered as taboo, which may lead to difficulties in accessing and using support services [29, 31]. It was claimed, that there is a general lack of public awareness of dementia and sensibilization while de-stigmatization of people with dementia might be helpful [6, 26, 36, 40]. This could prevent the trivialisation of the disease and thus facilitate the use of formal care [6]. Public education was suggested to help overcome stigmatization in dementia [20, 33, 40]. Important hindering aspects were the loss of autonomy and the ability of decision-making as well as the fear of being a burden to the family [38]. It was also mentioned that early stages of dementia were often considered as part of normal ageing [38]. Even healthcare professionals stigmatize people with dementia by giving medical problems more attention than psychological problems or by always using the term "dementia"—regardless of whether it had been diagnosed or not [29]. Barriers to accessing dementia care for people from ethnic minorities were seen in the prevalence of stigmas in families and communities around dementia and receiving care from people other than family members [41].

The dementia care nurses sometimes experience a refusal of their support because of their dementia-specific name. Therefore, non-dementia-specific names might support access to care.

### Early planning of formal care

*Early planning of formal care* was addressed in four studies [6, 20, 26, 35]. At the first suspicion of dementia, it should be planned with the person with dementia, how to proceed in the future regarding illness and health [20]. Initiating early contact with health-care professionals was also encouraged [6, 35]. The first contact was described as a challenge for further cooperation [35]. Early contact between health care professionals and families could be seen as a condition for care to be demand-oriented rather than an intervention in a sudden crisis [6].

In contrast, dementia care nurses often found that families of people with cognitive impairments did not contact professionals until the later stages of the dementia. They advocate that proactive contact with professionals could seldom help to overcome this barrier towards timely professional support. This experience was confirmed by general physicians in an Irish study [26].

### Similarities and differences between the professional perspective and the perspective of people with dementia and their informal carers

For a comparison of the professional perspective and the perspective of people with dementia and their informal carers, we used the scoping review by Bieber et al. [7]. This review also provides an overview of the influencing aspects of the access to and utilisation of formal community care in dementia, but focuses on the perspectives of people with dementia and informal carers [7]. An overview of the identified aspects found by Bieber et al. [7] compared to our findings is presented in Table 4.

**Table 4:** Overview of influencing aspects: professional perspective vs. people with dementia and their carers

Global themes and subthemes		Professionals	People with dementia/ informal carer [7]
**Aspects related to the individuals involved**			
**People with dementia and their informal carers**			
Ethnicity		X	X
Region of residence		X	X
Attitudes, expectations and experiences towards formal care and dementia		X	X
Family situation and social background		X	X
* Sociodemographic characteristics of people with dementia*			X
* Psychosocial aspects*			X
* Strategies to facilitate the use of services*			X
* Educational level of informal carers and people with dementia*			X
**Professionals**			
Competence of the professionals		X	X
Time resources of physicians		X	
Perceptions and attitudes of the healthcare professionals		X	
**Professionals, people with dementia and their informal carers**			
Relationship between professionals and people with dementia and their family		X	X
**Aspects related to the health and social care systems**			
Structures and complexity of the healthcare system	X		X
Financial aspects of the healthcare system	X		X
Multi-professional and interdisciplinary cooperation between institutions, service providers and professionals	X		
Coordinating care by persons or institutions	X		X
**Overarching aspects**			
Information about dementia and support services	X		
Stigmatization and public awareness	X		X
Early planning of formal care	X		

Bieber et al. [7] identified a total of 94 studies, while we included 29 publications that met our inclusion criteria. Two-thirds of the study population in the study by Bieber et al. [7] could be identified as informal carers, only one third could be identified as people with dementia. This demonstrates that research focusing on the influences of access to and utilisation of support services predominantly targets the perspectives of informal carers. In addition, lots of influencing aspects could be detected. Only one overarching aspect *was described* from the perspective of people with dementia and informal carers: *stigmatization and public awareness*, while the latter was not particularly addressed [7]. Views of study participants with dementia or informal carers mentioned one phenomenon, which can lead to stigmatization, namely that memory loss is often seen as a normal process of aging. The professional view was similar to this phenomenon [38] but there were more aspects about stigmatization, such as seeing dementia as a taboo [31, 36, 40, 41]. While health care professionals included in the current research stated that there is a general lack of public awareness or education in the communities [6, 36, 40], the study population reported by Bieber et al. [7] did not designate the need for further public awareness.

Aspects related to the *structures and complexity of the healthcare system* were less focused in the study by Bieber et al. [7] in comparison with the present scoping review.

While comparing these two reviews, we could also detect major similarities. Topics such as *ethnicity* or *attitudes, expectations and experiences towards formal care and dementia* were also addressed in our present review, but not as often as in the study by Bieber et al. [7]. On the other hand, Bieber et al. found out more details on these influencing aspects [7]. For example, they found, that some informal carers might have an estimation of their personal care competence, which can influence the decisions for or against the use of formal care services [7]. Other similarities, for example, were subthemes such as *region of residence*, *competencies of the professionals* or *family situation and social background*. Furthermore, the coordination of care as a demand both for people with dementia and their informal carers [7] as well as for professionals was a subject of a great discussion.

Another major common finding of both reviews is that both people with dementia and their informal carers either denounced the poor coordination and communication between service providers [7], or called for improved coordination of formal care, especially by healthcare professionals. Professionals mentioned several ways to improve this problem, such as having a single coordinating person or institution to manage the coordination of formal services [22, 24, 26, 29, 31]. Informal carers often felt that primary care physicians had sufficient knowledge about the selection of services available, but were not capable of coordinating care [7].

Both reviews also addressed the *relationship between professionals and people with dementia and their families*. However, while this was mentioned rather casually by people with dementia or informal carers [7], the topic was addressed in greater detail by professionals. For example, they assume that a trusting and positive relationship between the families and the healthcare professionals would have a positive effect on the continuing use of formal support services [6, 27, 35, 41].

The *competencies of professionals* have been described in detail in the current research and were mentioned in most of the included studies. This was also identified from the perspective of people with dementia and informal carers, but in less detail. For them, the knowledge of health care professionals was more important than their own personal skills, their social competencies, education or their knowledge in dementia issues which was considered in our present review. Professionals often requested further or specified training [20, 26, 29, 32, 34, 36, 39] as well as the improvement of their personal knowledge [19, 30, 36, 37, 40, 43].

The *family situation and social background* of the people with dementia was described in much more detail and variety by professionals than by people with dementia and informal carers [7], for example the relief of family carers when formal carers provide support. Both perspectives perceived that limited financial means of families with people with dementia can be a burden to the use of formal care services. Another aspect of the social background identified by Bieber et al. [7] was people with dementia living alone. They found out that *living alone* can be a significant predictor of receiving less formal care and that there are major differences in the type and extent of support for those people, depending on the country and the healthcare system in which they live. People with dementia *living alone* as their *social background* was a topic which was not addressed in the included publications of the current research.

Further aspects found in the study by Bieber et al. [7], which were not addressed in our included studies, were the influence of *sociodemographic characteristics*, including *gender related aspects* and the *state of health of the people with dementia*.

In contrast to our scoping review, the results from Bieber et al. [7] showed that the limited time resources of healthcare professionals had not been considered as an influencing aspect from the perspectives of people with dementia and their informal carers. Furthermore, the early planning of care was not reported by Bieber et al. [7]. Also, a *multi-professional and interdisciplinary cooperation between institutions, service providers and professionals*, which was considered by professionals to be beneficial and whose improvement was repeatedly called for, was not mentioned as an influencing factor by the people with dementia and their informal carers in the study by Bieber et al. [7].

## Discussion

This scoping review includes 29 studies that address influencing aspects of access to and use of formal care in dementia from a professional perspective. We structured the identified range of influencing aspects in *aspects related to the individuals involved*, *aspects related to the health and social care system* and *overarching aspects*. We discussed the findings with dementia care nurses, trained at our institute. A critical appraisal was conducted using the MMAT tool. The studies covered different types of health and social care services, but services for diagnostic and treatment in dementia were particularly often investigated.

The influences on the individual level of people with dementia and informal carers were related to sociodemographic aspects (i. e. ethnicity, region of residence and family situation) and regarding the professionals to their competencies and resources. Perceptions and attitudes were described for all involved parties. These psychosocial aspects were also highlighted in the review of influencing aspects from the perspective of people with dementia and informal carers. Especially the relationship between professionals and people with dementia and their families were highlighted in both reviews and approved within the consultation step with the dementia care nurses. This was confirmed in a systematic review examining communication between people with dementia and professionals [44]. Therefore, a positive relationship between the person with dementia and the formal carer can have a positive effect on their communication. Competencies of different professionals was a frequently investigated influence on access to and use of formal care in dementia in our review and was also mentioned in the review by Bieber et al. [7]. In particular, physicians (including specialists) and nurses, but also other professions such as social workers, therapists or psychologists unanimously emphasised that it is crucial that the different professions specialise and broaden their competencies. These competencies include dementia expertise, social and cultural skills and coordinating abilities. Several studies have found a lack of dementia specific knowledge [45, 46]. On the other hand, knowledge about efficacious dementia education programs is available, e. g. programs have to be relevant to participants’ role and experience or support practice-based learning [47]. Dementia specific training and education has improved in recent times. Nevertheless, there seems to be a lack of dementia-specific knowledge. It has to be asked, whether the education and training offers are available for the target groups or whether there are other reasons for the persistent lack of knowledge.

In the global topic *Aspects related to health- and social care systems,* the aspect *Coordinating care by persons or institutions* was frequently addressed. Several of our included studies suggested that a coordinating role or institution that helps people with dementia and their informal carers to orient in the health and social care system could be useful for the access and use of formal care services [6, 16, 24, 28, 29, 31, 32, 35, 40]. One of the key findings of the transnational research project Actifcare (Access to Timely Formal Care) is that a constant key contact person may be an important facilitator for the use of formal care services [6, 31]. One single named health or social care professional who is responsible for coordinating care, e.g. a case-manager, is also considered useful according to the current NICE guideline [48] regarding dementia. Moreover, a meta-analysis investigating the effectiveness of coordinating interventions in dementia care concluded that such interventions can have a positive impact on people with dementia and their informal carers [49]. In the majority of instances, the role for coordinating or planning formal support is taken over by the general practitioner as the first point of contact. However, physicians often have limited time to devote to adequate care planning and organisation for people with dementia [17, 18, 24, 32]. Instead, a dedicated resource consisting of a professional with dementia-specific training, such as a dementia care nurse, would be suitable for this coordinating position. A comparable use of a person, a so-called care navigator, who coordinates the support and care of people with dementia, is recommended by Bernstein et al. [50]. Integrated care is a further approach to a cross-sectoral, holistic and comprehensive care concept, which includes case-management [51, 52].

Generally, in numerous studies included in our scoping review, *multi-professional and interdisciplinary cooperation between social and health care professions* as well as *institutions* has always been considered to be beneficial [18, 21, 23–25, 29, 30, 35]. In the scoping review about the non-professional perspective [7], multi-professional cooperation between health professionals was hardly addressed, and if so, it was in the context of well-coordinated care. We assume that the main cause for this may be the limited insights of people with dementia or informal carers into the professional’s environment and the scope of their responsibilities. Several publications have concluded that collaborative care by multiple healthcare providers can have a positive effect on people with dementia and have suggested further enhancement [53–55].

In the global topic *Overarching Aspects,* the aspect *information about dementia and support services* was frequently addressed [6, 20, 29–33]. Both informative and educational services for people with dementia as well as for their informal carers were considered to be beneficial in accessing formal support. Experts in the Actifcare study [31] suggested setting up an informative website. An example of the realization of this kind of website, which aims to provide information, (online) education and information about regional service providers, can be found in *DigiDem*, a pilot project in Bavaria, Germany, which is currently in the process of implementation [56, 57]. Stigmatization as a barrier for accessing and using formal care in dementia was mentioned in one third of the studies included in our review [6, 20, 26, 29, 31, 33, 34, 36, 40, 41]. A review concerning dementia-related stigmatization research found stigmatization attitudes in some health providers, but few evidence-based stigma decreasing approaches [15]. We found only a few studies investigating early planning of formal care, although it was seen as a precondition for need-driven care [6]. This topic was not mentioned in the review related to the perspective of people with dementia and informal carers. That could be interrelated with a predominant late search for formal care in dementia [4].

Our review shows that predominantly physicians were involved in investigations of access to and use of formal care in dementia. Studies investigating different professional backgrounds could be identified. For further research, the question should be which professions have a close contact to the target groups and can therefore give deep insights in the motivations and reasons to search for or to refuse formal support and care.

To understand the complexity of access to and use of formal care in dementia it is necessary to consider the various influences on an individual and system-related level, as well as overarching aspects. All perspectives should be taken into consideration to enhance the understanding for the influences on formal care use in dementia and to find ways to facilitate access to formal services. Good practice examples, like the dementia care nurses or the Dementia Care Managers, should be implemented in standard care structures [15, 58]. The dementia care nurses, involved in the consultation step of this review, confirmed that this professional role is appropriate to support people with dementia and informal carers when looking for timely and tailored formal care. However, the dementia care nurses are not yet part of standard care, but initial steps are being taken to find ways for them to become part of the regular health and social care system in Germany.

Against the background of the pandemic caused by COVID-19, local infection control restrictions are a significant challenge for people with dementia and their relatives [59–61]. One problem is the risk of inadequate care due to bans on visits imposed by authorities, such as physiotherapists or other specialists [60]. Additionally, professionals can increase the risk of virus transmission by providing care in close proximity to the person with dementia [62]. Cuffaro et al. [59] recommend telemedical concepts, such as web-based training courses for informal carers or digital psycho-educational services. Access to and the use of telemedical concepts were not mentioned in the studies included in our review. Further research should consider the potential of technology-based interventions to improve access to formal dementia care.

### Strengths and Limitations

Strengths of this scoping review are the conduct of a critical appraisal of the included publications, and the discussion of the findings with the dementia care nurses. The literature suggests critical appraisal and consultation exercises as facultative steps of scoping reviews [10, 13]. Above all, the discussion with the dementia care nurses guided us to understand the influence of several aspects in providing formal care considering the German health and social care system.

A limitation of this review was the search for literature in only one database (Medline). At this point the literature search could have been extended to databases such as CINAHL, PsychInfo, Embase or Social Science Citation Index. The use of more than one database and the search for grey literature is also in line with the recommendations of the methodology paper by Khalil et al. [9]. Only certain aspects could be selected for the discussion, as a complete consideration of all aspects would have gone beyond the scope of this paper. The comparison of the perspectives of professionals, people with dementia and informal carers includes a selection of influencing aspects. There may be other influencing aspects, which should have been taken into account.

## Conclusion

The perspectives of professionals showed some similarities, but also differences to the views of informal carers and people with dementia. While the latter increasingly addressed more psychosocial aspects [7], the health care professionals often referred to systemic and structural barriers or supporting aspects within the health care system. In contrast to physicians, the perspectives of nurses on access to and use of formal dementia support and care have rarely been examined. Therefore, we conclude that nurses and other health and social care professions should be given more consideration in dementia care research. In addition, we consider the expansion of further training for health care professions to be appropriate in order to ensure high expertise in dementia care. Furthermore, coordinated dementia-specific care is necessary to provide adequate support for people with dementia and their families. Several health professions should be involved. We suggest that professionals with dementia-specific training such as Dementia Care Nurses or community care nurses as well as case managers who are specialised in dementia care could take on this coordinating role. This in return, could relieve other professionals such as general practitioners. In this context, it would be crucial that tasks and responsibilities are clearly defined and distributed.

## Data Availability

Additional data used during the current study are available from the corresponding author in response to reasonable requests.

## Abbreviations

Actifcare: Access To Timely Formal Care
MMAT: Mixed Methods Appraisal Tool
